# Prospective analysis of *UGT1A1* promoter polymorphism for irinotecan dose escalation in metastatic colorectal cancer patients treated with bevacizumab plus FOLFIRI as the first-line setting: study protocol for a randomized controlled trial

**DOI:** 10.1186/s13063-016-1153-3

**Published:** 2016-01-25

**Authors:** Yung-Sung Yeh, Hsiang-Lin Tsai, Ching-Wen Huang, Jui-Ho Wang, Yi-Wen Lin, Hsiu-Chih Tang, Yung-Chuan Sung, Chang-Chieh Wu, Chien-Yu Lu, Jaw-Yuan Wang

**Affiliations:** Division of Trauma, Department of Surgery, Kaohsiung Medical University Hospital, Kaohsiung Medical University, Kaohsiung, Taiwan; Division of Gastroenterology and General Surgery, Department of Surgery, Kaohsiung Medical University Hospital, Kaohsiung Medical University, Kaohsiung, Taiwan; Graduate Institute of Clinical Medicine, College of Medicine, Kaohsiung Medical University, Kaohsiung, Taiwan; Division of General Surgery Medicine, Department of Surgery, Kaohsiung Medical University Hospital, Kaohsiung Medical University, Kaohsiung, Taiwan; Graduate Institute of Medicine, College of Medicine, Kaohsiung Medical University, Kaohsiung, Taiwan; Department of Surgery, Faculty of Medicine, College of Medicine, Kaohsiung Medical University, Kaohsiung, Taiwan; Division of Colorectal Surgery, Department of Surgery, Kaohsiung Medical University Hospital, Kaohsiung Medical University, Kaohsiung, Taiwan; Division of Colorectal Surgery, Department of Surgery, Kaohsiung Veterans General Hospital, Kaohsiung, Taiwan; Department of Surgery, Tainan Municipal Hospital, Tainan, Taiwan; Colon and Rectal Surgery, Tainan Sin-Lau Hospital, Tainan, Taiwan; Division of Hematology-Oncology, Department of Internal Medicine, Cathay General Hospital, Taipei, Taiwan; Division of Colon and Rectal Surgery, Department of Surgery, Tri-Service General Hospital, National Defense Medical Center, Taipei, Taiwan; Division of Gastroenterology, Department of Internal Medicine, Kaohsiung Medical University Hospital, Kaohsiung Medical University, Kaohsiung, Taiwan; Department of Internal Medicine, Faculty of Medicine, College of Medicine, Kaohsiung Medical University, Kaohsiung, Taiwan; Center for Biomarkers and Biotech Drugs, Kaohsiung Medical University, Kaohsiung, Taiwan

**Keywords:** *UGT1A1*, Bevacizumab, FOLFIRI, Dose escalation, Metastatic colorectal cancer

## Abstract

**Background:**

Irinotecan is approved and widely administered to metastatic colorectal cancer (mCRC) patients; however, it can cause severe toxicities including neutropenia and diarrhea. The polymorphisms of genes encoding drug-metabolizing enzymes can play a crucial role in the increased susceptibility of cancer patients to chemotherapy toxicity. Therefore, we plan to explore the effect of the genetic polymorphism of uridine diphosphate glucuronosyltransferase 1A1 (*UGT1A1*) for irinotecan detoxification in mCRC patients. This trial will compare the clinical outcomes and side effects observed in mCRC patients treated with bevacizumab plus 5-fluorouracil/leucovorin/irinotecan (FOLFIRI) with and without *UGT1A1* genotyping and irinotecan dose escalation. A total of 400 mCRC patients were randomized into a study group and a control group.

**Methods/Design:**

This trial is a prospective, multicenter, randomized clinical trial comparing *UGT1A1* promoter polymorphism for irinotecan dose escalation in mCRC patients administered with bevacizumab plus FOLFIRI as the first-line setting. The enrolled patients were randomly assigned to one of two groups, a study group and a control group, on the basis of receiving *UGT1A1* genotyping or not. The study group receive a biweekly FOLFIRI regimen, with irinotecan dose escalation based on *UGT1A1* genotyping; whereas the control group receive the conventional biweekly FOLFIRI regimen without *UGT1A1* genotyping. The clinicopathological features, response rates, toxicity, and progression-free survival or overall survival will be compared between the two groups.

**Discussion:**

Patients with mCRC undergoing *UGT1A1* genotyping may receive escalated doses of irinotecan for a potentially more favorable clinical response and outcome, in addition to comparable toxicities. Such personalized medicine based on genotyping may be feasible for clinical practice.

**Trial registration:**

NCT02256800. Date of registration: 3 October 2014. Date of first patient randomized: 16 January 2015

## Background

### Rationale for the trial

Metastatic lesions are reported in 20–25 % of patients with an initial diagnosis of colorectal cancer (CRC) and in up to 50 % of patients with an eventful treatment course. Because of the limited treatment response of metastatic colorectal cancer (mCRC) patients to 5-fluorouracil (5-FU) combined with leucovorin (LV), other therapeutic agents with different mechanisms can be considered, such as irinotecan, a potent inhibitor of topoisomerase I involved in unwinding the DNA during replication [1–4]. Bevacizumab, the first antiangiogenic agent approved for cancer treatment, is a humanized monoclonal antibody that inhibits tumor angiogenesis by blocking vascular endothelial growth factor [5, 6].

### Previous trials

Infusional FU or LV plus an irinotecan-based regimen (FOLFIRI) with bevacizumab has been widely used as a first-line treatment for patients with mCRC [1]. Recently, a prospective analysis of uridine diphosphate glucuronosyl transferase 1A1 (*UGT1A1*) genotyping was reported to guide irinotecan dose escalation (FOLFIRI regimen) in combination with biweekly bevacizumab in a first-line treatment setting of mCRC patients and resulted in satisfactory therapeutic outcomes clinically [7]. Our previous retrospective study showed that the clinical response rate of patients with mCRC treated with FOLFIRI plus bevacizumab under *UGT1A1* genotyping and irinotecan dose escalation was significantly higher than that of those without *UGT1A1* genotyping and dose escalation [8]. The two groups (conventional and escalated doses of irinotecan after *UGT1A1* genotyping) did not differ statistically regarding receiving prior surgery or the subsequent administration of 5-FU/LV/oxaliplatin adjuvant chemotherapy. The clinical response rate was significantly higher in the mCRC patients receiving *UGT1A1* genotyping and irinotecan dose escalation in advance than in those not receiving genotyping. The two groups did not differ significantly in grade 3/4 adverse events. Furthermore, the progression-free survival (PFS) was significantly higher in the patients with mCRC who received *UGT1A1* genotyping and irinotecan dose escalation before systemic treatment than in those who did not receive prospective *UGT1A1* genotyping, with a median PFS of 12.2 months versus 9.4 months [8].

A prodrug that is converted into the active metabolite 7-ethyl-10-hydroxycamptothecin (SN-38), irinotecan, is potently toxic to topoisomerase I in vivo, thus interrupting DNA replication in cancer cells and resulting in a high rate of cell death. SN-38 is further detoxified into its inactive metabolite, SN-38G, through glucuronidation by the enzyme uridine diphosphate glucuronosyltransferase (UGT) in the liver. The glucuronidation of SN-38 to SN-38G is the decisive step in the metabolism and detoxification of irinotecan. The number of repeats in the TATA box of the *UGT1A1* promoter alters *UGT1A1* activity, with six TA repeats representing the most common allele of the *UGT1A1* gene (UGT1A1*1, wild-type) and seven TA repeats representing a variant allele (UGT1A1*28, mutant type).

Reduced gene transcription and expression of *UGT1A1* are observed in individuals with the UGT1A1*28 variant; consequently, reduced SN-38 glucuronidation and increased irinotecan-related toxicity are well-established in mCRC patients with the UGT1A1*28 variant. UGT1A1*28 is considered the main predictor of toxicity in mCRC patients treated with irinotecan. The use of *UGT1A1* genotyping as a predictive marker of irinotecan-induced severe neutropenia or diarrhea has been approved by the Food and Drug Administration of the United States [2]. However, no predictive marker exists for irinotecan-based chemotherapy in the treatment of mCRC. Hence, we will evaluate the efficacy and safety profile of FOLFIRI plus bevacizumab when the irinotecan dosage is adjusted on the basis of the blood *UGT1A1* genotype through a prospective, multicenter, randomized controlled study.

### Objective

The primary objective is to implement a prospective, randomized multicenter trial to evaluate the efficacy of the FOLFIRI-plus-bevacizumab regimen with the irinotecan dosage adjusted on the basis of the *UGT1A1* genotype as measured by the PFS at 1 year. The secondary objective is to evaluate the efficacy of the FOLFIRI-plus-bevacizumab regimen with the irinotecan dosage adjusted on the basis of the *UGT1A1* genotype as measured by the objective response rate (ORR) and toxicities, and finally to measure overall survival (OS).

### Hypothesis

On the basis of our previous study, we hypothesize that patients with mCRC who receive pretherapeutic *UGT1A1* genotyping and subsequent irinotecan dose escalation can achieve more favorable responses and outcomes without a significant increase in toxicity while using the FOLFIRI-plus-bevacizumab regimen.

### Trial sites

The trial will be performed at 10 sites of the Colorectal Cancer Consortium, part of the Grant of Biosignature in Colorectal Cancers of the Academia Sinica in Taiwan. Most of these sites have participated in previous randomized controlled trials, and all of the centers are adequately trained and prepared according to “Good Clinical Practices” elaborated by the International Conference on Harmonization of Technical Requirements for Registration of Pharmaceuticals for Human Use to participate in this trial.

## Methods/Design

### Description of the study

**Fig. 1:**
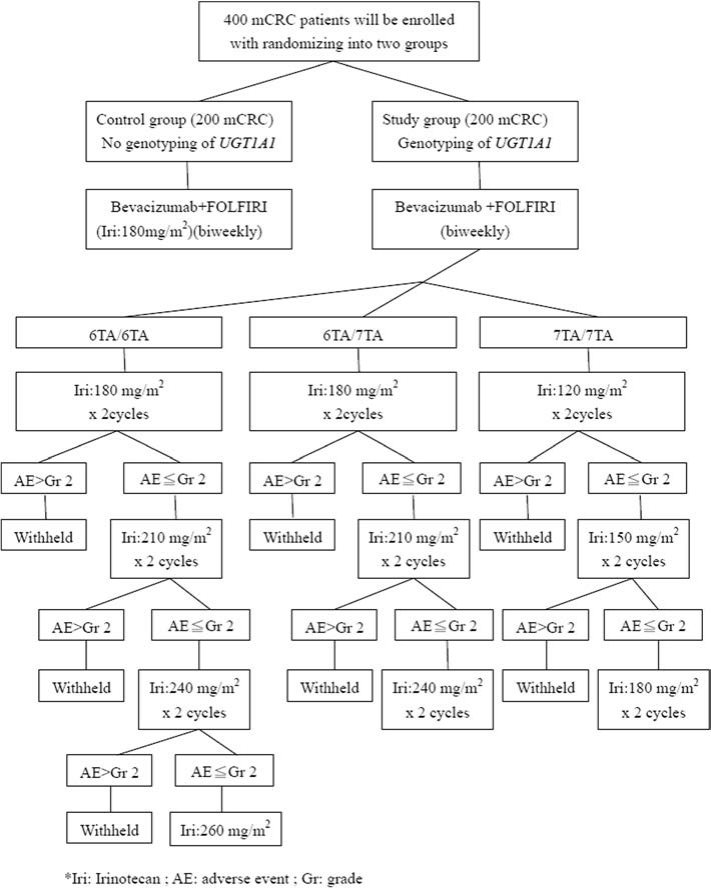
Flowchart of this trial. The patients enrolled in the control group will be treated with the conventionally recommended dose of irinotecan without *UGT1A1* genotyping in advance. The patients enrolled in the study group will be divided further into three subgroups according to their *UGT1A1* genotypes; irinotecan dose escalation will be based on *UGT1A1* genotyping

The details of this study are provided in Table 1. This trial is a multicenter, randomized, controlled clinical trial comparing *UGT1A1* promoter polymorphism for irinotecan dose escalation in mCRC patients administered with FOLFIRI plus bevacizumab as the first-line setting. The treatment administrators or patients are not masked to treatment allocation. In October 2014 400 mCRC patients were randomly enrolled into the two groups. The study arms are described as follows (Fig. 1):

**Table 1:** Schedule of assessments

Assessment/Procedure	Before enrollment (screening)	Clinical regimen		Clinical tumor assessment	Completion/Early termination visit	Survival follow-up
Available data will be collected; no additional diagnostic or monitoring procedures shall be applied to the patients other than routine clinical practice.						
Study week		Every 2 weeks	Every 4 weeks	Every 12 weeks		Every 12 weeks
Informed consent	x					
Demographics and medical history	x					
Cancer treatment history	x					
Urinalysis	x	x			x	
Tumor assessment	x			x	x	x
ECOG performance status	x	x			x	
Hematology	x	x			x	
Clinical chemistry	x	x			x	
Creatinine clearance (calculated)	x	x				
Physical examination and vital signs	x	x			x	
Weight and height	x	x				
Concomitant medications	x	x			x	
Adverse events	x	x	x	x	x	x
Study drug administration	x	x				
Survival and tumor status/other anticancer treatment						x

### Control group

The patients enrolled in the control group will be treated with the conventionally recommended dose of irinotecan, without *UGT1A1* genotyping in advance. The regimen for the treatment will consist of bevacizumab (5 mg/kg as a 120-minute intravenous (IV) infusion) on day 1, followed by irinotecan (180 mg/m^2^ as a 120-minute IV infusion), LV (200 mg/m^2^ as an IV infusion over 2 hours), and 5-FU (2800 mg/m^2^ as an IV infusion over a 46-hour period) and will be repeated biweekly. The control group will be *UGT1A1* genotyped at the end of the trial.

### Study group

The patients enrolled in this group were further divided into three subgroups on the basis of their *UGT1A1* genotypes:

- *Subgroup 1*: *UGT1A1* 6TA/6TA genotype
- The treatment regimen will comprise bevacizumab (5 mg/kg as a 120-minute IV infusion) on day 1, followed by irinotecan (180 mg/m^2^ as a 120-minute IV infusion), LV (200 mg/m^2^ as an IV infusion over 2 hours), and 5-FU (2800 mg/m^2^ as an IV infusion over a 46-hour period) and will be repeated biweekly. The adverse effects (AEs), hematological or non-hematological, will be observed after two cycles of each dose of irinotecan. If the AEs are below grade 2, the dose will be gradually escalated in steps of 30 mg/m^2^. The estimated maximal dose of irinotecan is 260 mg/m^2^.
- *Subgroup 2*: *UGT1A1* 6TA/7TA genotype
- The treatment regimen will comprise bevacizumab (5 mg/kg as a 120-minute IV infusion) on day 1, followed by irinotecan (180 mg/m^2^ as a 120-minute IV infusion), LV (200 mg/m^2^ as an IV infusion over 2 hours), and 5-FU (2800 mg/m^2^ as an IV infusion over a 46-hour period) and will be repeated biweekly. The irinotecan dose will be increased in increments of 30 mg/m^2^ (180, 210, 240) every two cycles in the absence of grade 2 or worse AEs. The maximal dose of irinotecan will be 240 mg/m^2^.
- *Subgroup 3*: *UGT1A1* 7TA/7TA genotype
- The treatment regimen will comprise bevacizumab (5 mg/kg as a 120-minute IV infusion) on day 1, followed by irinotecan (120 mg/m^2^ as a 120-minute IV infusion), LV (200 mg/m^2^ as an IV infusion over 2 hours), and 5-FU (2800 mg/m2 as an IV infusion over a 46-hour period) and will be repeated biweekly. After two cycles of each irinotecan dose, hematological and non-hematological AEs will be observed. If the toxicities are below grade 2, the dose will be gradually escalated in steps of 30 mg/m^2^. The estimated maximal dose of irinotecan is 180 mg/m^2^.

### Number of patients

A total of 400 mCRC patients histologically proved to have adenocarcinoma in primary CRC, either metachronous or synchronous, were enrolled in the study.

### Randomization

The participants were randomized using sealed, opaque, individually numbered envelopes, with the restriction of selecting one per person. The envelopes contained data sheets with information on group allocation and the randomization number.

### Target population/inclusion and exclusion criteria

## Inclusion criteria

1. Patients had to be older than 20 years and have a life expectancy of more than 3 months
2. Absence of other primary malignancies
3. Absence of central nervous system (CNS) metastases
4. No major underlying diseases (such as cardiovascular, cerebrovascular, malignant hypertension, inadequate hematological function, kidney, liver, or other major diseases) or any active infections
5. mCRC confirmed in reports from pathologists or radiologists according to the Response Evaluation Criteria in Solid Tumors (RECIST) criteria
6. An Eastern Cooperative Oncology Group (ECOG) performance status of 0–2
7. Subjects were willing to sign an informed consent form
8. Subjects had to be at least 28 days from their most recent surgery or have wounds that were healed
9. Female participants who were not postmenopausal (less than 12 months of amenorrhea) or surgically sterile had to agree to use a highly effective contraceptive method (i.e., with a failure rate of <1 % per year, such as sexual abstinence, hormonal implants, combined oral contraceptives, or a vasectomized partner) during the treatment period and for at least 6 months after the last dose of the study drug. If it was not possible to use a highly effective contraceptive method, two barrier methods were to be used
10. Male participants had to agree to use a highly effective contraceptive method (i.e., with a failure rate of <1 % per year, such as a vasectomy, sexual abstinence, or a female partner using hormonal implants or combined oral contraceptives) during the trial and for a period of at least 6 months after the last dose of the study drug. If it was not possible to use a highly effective contraceptive method, two barrier methods were to be used

## Exclusion criteria

1. Patients who did not meet the inclusion criteria or who were unwilling to participate
2. Prior or current antiangiogenic treatment
3. Treatment with any other investigational agent within 28 days prior to enrollment in this study
4. Inadequate hematological function, as indicated by all of the following:
  Absolute neutrophil count <1.5 × 10^9^/L
  Platelet count <100 × 10^9^/L
  Hemoglobin <9 g/dL
5. Inadequate liver function, as indicated by all of the following:
  Total bilirubin ≥1.5 × upper limit of normal (ULN)
  Aspartate transaminase (AST) and alanine aminotransferase (ALT) ≥2.5 × ULN; in patients with liver metastasis, AST and ALT ≥3.0 × ULN
  Alkaline phosphatase ≥2.0 × ULN
6. Inadequate renal function, as indicated by all of the following:
  Serum creatinine >1.25 × ULN or calculated creatinine clearance <50 mL/min
  Urine dipstick for proteinuria at least 2+ unless a 24-hour urine protein <1 g of protein is demonstrated
7. Inadequately controlled hypertension (defined as systolic blood pressure >160 mmHg and/or diastolic blood pressure >100 mmHg)
8. Prior history of hypertensive crisis or hypertensive encephalopathy
9. History or evidence upon physical or neurological examination of a CNS disease (e.g., seizures) unrelated to cancer unless adequately treated with standard medical therapy
10. Significant vascular disease (e.g., aortic aneurysm requiring surgical repair or recent arterial thrombosis) within 6 months of study enrollment
11. Any previous venous thromboembolism with less than National Cancer Institute Common Terminology Criteria for Adverse Events (NCI-CTCAE) grade 3
12. Untreated CNS metastases or treatment of brain metastases, either by surgical or radiation techniques, had to be completed more than 4 weeks prior to the first study treatment
13. History of hemoptysis of at least grade 2 (defined as ≥2.5 mL of bright-red blood per episode) within 1 month of study enrollment
14. History or evidence of inherited bleeding diathesis or significant coagulopathy at the risk of bleeding (i.e., in the absence of therapeutic anticoagulation)
15. Current or recent (within 10 days of study enrollment) use of aspirin (>325 mg/day) or clopidogrel (>75 mg/day); current or recent (within 10 days prior to first dose of bevacizumab) use of therapeutic oral or parenteral anticoagulants or thrombolytic agents for therapeutic purposes
  Note: the use of full-dose oral or parenteral anticoagulants was permitted as long as the international normalized ratio (INR) or partial thromboplastin time (PTT) was within therapeutic limits (according to the medical standard of the institution) and the patient had been on a stable dose of anticoagulants for at least 2 weeks at the time of study enrollment. The prophylactic use of anticoagulants was allowed
16. Surgical procedure (including open biopsy, surgical resection, wound revision, or any other major surgery involving entry into a body cavity) or significant traumatic injury within 28 days prior to study enrollment or anticipation of the need for major surgical procedure during the course of the study
17. History of abdominal fistula, gastrointestinal (GI) perforation, intra-abdominal abscess, or active GI bleeding within 6 months prior to the first study treatment
18. Serious, non-healing wound, active ulcer, or untreated bone fracture
19. Known hypersensitivity to any component of bevacizumab or any of the study drugs
20. Active infection requiring intravenous antibiotics at the time of the first study treatment
21. Other malignancy within 5 years prior to study enrollment, except for localized cancer in situ such as basal or squamous cell skin cancer
22. Evidence of any other disease, neurological or metabolic dysfunction, abnormal physical examination finding, or laboratory finding leading to a reasonable suspicion of a disease or condition that contraindicated the use of any of the study drugs, placed the patient at a higher risk for treatment-related complications, or may have affected the interpretation of the study results
23. Requirement for treatment with any medicinal product that contraindicated the use of any of the study drugs, may have interfered with the planned treatment, affected patient compliance, or put the patient at a high risk for treatment-related complications

### Genotyping

For analyzing constitutional gene polymorphisms, DNA was first extracted from 4 mL of peripheral blood using a PUREGENE® DNA isolation kit (Gentra Systems, Inc., Minneapolis, MN, USA). The genomic DNA from the patients was then analyzed using direct sequencing to determine the *UGT1A1* promoter region genotype. The primers used in this study were designed using primer 3 free software (http://primer3.wi.mit.edu). The sequences of the forward and reverse primers were 5ʹ-AGTCACGTGACACAGTCAAACA-3ʹ and 5ʹ-CTTTGCTCCTGCCAGAGGTT-3ʹ, respectively. The polymerase chain reaction (PCR) reaction volume was 40 μL, and the PCR conditions for the glutathione *S*-transferase pi 1 (*GSTP1*) were as follows: 94.0 °C for 5 minutes; 30 cycles of denaturation for 30 seconds at 94.0 °C; annealing for 20 seconds at 67.5 °C; primer extension for 20 seconds at 72.0 °C; and final extension for 10 minutes at 72.0 °C. A fragment analysis of the PCR products was conducted to verify the genotypes by using the automated capillary electrophoresis on the ABI PRISM 310 Genetic Analyzer (Applied Biosystems, Foster City, CA, USA), and the genotypes were analyzed using GeneScan and Genotyper software (Applied Biosystems, Foster city, CA, USA).

### Interventions and trial timeline

The recruitment period of this study is expected to last 24 months. The trial’s primary objective is expected to be attained within 36 months. The survival data will be collected until death or a patient’s request for withdrawal. The end of the study will be defined at 24 months after the last patient has enrolled.

### Efficacy and safety

## Efficacy outcome measures

Responses are assessed radiologically by using computed tomography scans, magnetic resonance images, bone scans, or positron emission tomography scans, with optimal responses being recorded. The time for assessing the first response is typically after the sixth cycle in patients who have received bevacizumab combined with FOLFIRI chemotherapy. The responses are classified by a radiologist according to RECIST Version 1.1 [9].

1. A complete response is defined as the disappearance of all target lesions
2. A partial response (PR) is defined as at least a 30 % decrease in the sum of the longest diameter, taking the baseline sum’s longest diameter as a reference point
3. Progressive disease is defined as at least a 20 % increase in the sum of the longest diameter of the target lesions, taking the smallest sum of the longest diameters recorded before the patient started to receive treatment as a reference. It can also be defined as the identification of one or more new lesions
4. Stable disease is defined as neither having sufficient shrinkage to quality as a PR nor a sufficient increase to qualify as a progressive disease
5. PFS is defined as the time from the beginning of treatment until the first documentation of progression, regardless of the patient’s treatment status
6. OS is defined as the time from the beginning of treatment until the date of a death event or the last recorded date of follow-up.

## Safety outcome measures

The AEs will be assessed in each cycle using the NCI-CTCAE 4.0 (http://ctep.cancer.gov/reporting/ctc.html,). AEs over grade 2 will be noted, such as the following:

1. Hypertension at least grade 3
2. Proteinuria at least grade 3
3. GI perforation, abscesses, and fistulae (any grade)
4. Wound healing complications at least grade 3
5. Hemorrhage at least grade 3 (CNS bleeding of any grade; hemoptysis at least grade 2)
6. Arterial thromboembolic events (any grade)
7. Venous thromboembolic events at least grade 3
8. Posterior reversible encephalopathy syndrome (any grade)
9. Congestive heart failure at least grade 3
10. Non-GI fistula or abscess at least grade 2
11. Cases of an elevated ALT or AST with or without elevated bilirubin (total bilirubin ≥2 mg/dL)
12. Suspected transmission of an infectious agent by the study drug

## Paragraph of dose reduction of irinotecan and stopping rule

Dose escalation was terminated if grade 3 or 4 AEs occurred, and when such grade 3 or 4 AEs did occur, the patients were treated subsequently with the highest dose of irinotecan they were able to tolerate previously. The treatment was stopped in the event of patient withdrawal, disease progression, or unacceptable toxic effects (non-hematological grade 4 toxicity, no recovery from grade 3 toxicity after two dose adjustments or non-recovery after a 2-week treatment delay). Any dose reduction was permanent.

## Statistical methods

### Determination of sample size

This study enrolled approximately 400 mCRC patients. It is anticipated that the PFS will increase by 2.8 months compared with the PFS expected when using the conventional irinotecan dose of 180 mg/m^2^; hence, these parameters were selected for calculating the study power. An initial power calculation suggested that a minimum of 200 patients were required in each group to achieve statistical significance with a power of 80 % at a 5 % significance level. It is estimated that approximately 20 % of the 400 mCRC patients will fail to complete the study. The enrolled patients were *UGT1A1* genotyped before therapy and then randomly assigned to one of the two groups, the control and study groups. The control group includes mCRC patients who receive the conventional FOLFIRI regimen. The patients in the study group will be provided dose escalation depending on the results of their genotyping.

All of the data were analyzed using the Statistical Package for the Social Sciences Version 17.0 software (SPSS, Inc., Chicago, IL, USA). The descriptive variables of the patient characteristics and toxicities were calculated directly from the database. A chi-squared test or Fisher’s exact test was used to compare the toxicities and responses between the two groups. The PFS and OS were calculated and plotted according to the Kaplan-Meier method and were compared using a log-rank test. A probability of less than 0.05 was considered statistically significant.

### Analysis of primary endpoints

The PFS will be analyzed to examine superiority between the two groups.

### Analysis of secondary endpoints

The efficacy of the bevacizumab-plus-FOLFIRI regimen with the irinotecan dosage adjusted on the basis of the *UGT1A1* genotype, as measured using the ORR and toxicities, will be evaluated. This ORR will include the response rates (confirmed complete and partial responders), estimated difference in response rates, and associated 95 % confidence intervals.

## Ethical approval

In compliance with the Helsinki Declaration, this study was approved by the Institutional Review Board of Kaohsiung Medical University Hospital, Kaohsiung Medical University, Kaohsiung, Taiwan. (IRB number: KMUHIRB-20130020). We will obtain informed consent from each participant.

## Discussion

Irinotecan is converted into SN-38 by a carboxylesterase and finally metabolized by the enzyme UGT (predominantly by the UGT1A1 isoenzyme). Recently, Marcuello et al. [10] conducted a genotype-directed dose-finding study on irinotecan in combination with fluorouracil/LV as a first-line treatment for advanced mCRC.

Genotyping and sequencing data have led to the discovery of over 100 variants within the promoter regions and coding sequence of the *UGT1A* genes [11]. Many of these variants exhibit allele frequencies of up to 40–50 % in the general population, which are in linkage disequilibrium. However, a few variants are of sufficient frequency in the general population to be classified as polymorphisms [11–13]. Polymorphisms lead to different degrees of transcriptional and functional alterations, which may reduce UGT activity and result in the pathology of the affected individuals [11, 14].

We conducted the current study, based on our previous retrospective investigation published in 2014 [8], of which we had the prospective, randomized trial to obtain Institutional Review Board (IRB) approval in Taiwan. At that time there was no relevant information regarding UGT1A1*6 genotyping that could be used for directing dose escalation of irinotecan (trade name: Camptosar® other name: Camptothecin-11(CPT-11)) in Taiwanese mCRC patients. In fact, one recent study published by Kim et al*.* in 2015 [15], Patients were genotyped for UGT1A1 and stratified according to the number of defective alleles (DA; *28 and *6) wherein they showed that the recommended doses were 300 (0 DA), 270 (1 DA) and 150 (0 DA) mg/m^2^. Conversely, there were no significant differences in the efficacy or toxicity of FOLFIRI between patients with the UGT1A1*1/*1 genotype and those with the UGT1A1*1/*6 or *1/*28 genotypes [16]. Additionally, Maeda et al. had demonstrate that UGT1A1*6 A/A polymorphism were not statistically different between the Caucasian population and Asian populations (*P* value = 1.0) [17].

Patients with homozygous polymorphism of the *UGT1A1* promoter (UGT1A1*28) were more frequently associated with severe toxicity following irinotecan treatment. The genotypes of the promoter polymorphism of the *UGT1A1* gene in our patients were either *UGT1A1* 6/6 or *UGT1A1* 6/7. Individuals who are homozygous for the UGT1A1*28 allele are at an increased risk of toxicity following the initiation of FOLFIRI treatment; a low initial dose should be considered for patients known to be homozygous for the UGT1A1*28 allele (seven repeats). Heterozygous patients (carriers of one variant allele and one wild-type allele, which results in intermediate *UGT1A1* activity) may be at an increased risk of toxicity; however, clinical results were variable, and such patients could tolerate the normal starting dose [18]. Furthermore, the possible role of bevacizumab as a protective agent against irinotecan toxicity in these patients should be investigated. The addition of bevacizumab improved the pathological response and protection against hepatic injuries in patients treated with oxaliplatin-based chemotherapy for colorectal liver metastases [19]. This trial is performed to demonstrate the prognostic advantage of *UGT1A1* genotyping and irinotecan dose escalation before systemic chemotherapy to patients with mCRC.

In our previous study, a combination of bevacizumab and FOLFIRI as a first-line therapy for mCRC patients was retrospectively demonstrated to have a favorable response rate and acceptable toxicity. Nearly 70 % of the patients (55 of 79) with mCRC who had undergone pretherapeutic *UGT1A1* genotyping and received an escalated dose of irinotecan responded clinically to bevacizumab-plus-FOLFIRI chemotherapy [8]. Therefore, we are prospectively conducting a clinical trial to demonstrate that irinotecan dose escalation can achieve more favorable responses and outcomes without a significant increase in toxicities while using the FOLFIRI-plus-bevacizumab regimen. The use of novel genomic DNA analysis in our current study can achieve more favorable clinical outcomes for mCRC patients.

## Trial status

The trial started in January 2015 and is expected to finish in December 2017. Sixty-eight patients, including 34 patients in the study group and 34 patients in the control group, had been enrolled into the study by the end of the November 2015.

## Abbreviations

5-FU: 5-fluorouracil
AEs: adverse effects
ALT: alanine aminotransferase
AST: aspartate transaminase
CNS: central nervous system
CRC: colorectal cancer
ECOG: Eastern Cooperative Oncology Group
FOLFIRI: chemotherapy of 5-fluorouracil/leucovorin/irinotecan
LV: leucovorin
mCRC: metastatic colorectal cancer
NCI-CTCAE: National Cancer Institute-Common Terminology Criteria
ORR: objective response rate
OS: overall survival
PFS: progression-free survival
PR: partial response
RECIST: Response Evaluation Criteria in Solid Tumors
SN-38: active metabolite, 7-ethyl-10-hydroxycamptothecin, which is potently toxic to topoisomerase I
ULN: upper limit of normal
UGT: uridine diphosphate glucuronosyltransferase
*UGT1A1*: uridine diphosphate glucuronosyltransferase 1A1
